# Knowledge, attitudes, and practices regarding COVID-19 among pharmacists partnering with community residents: A national survey in Japan

**DOI:** 10.1371/journal.pone.0258805

**Published:** 2021-10-26

**Authors:** Dan Kambayashi, Toshie Manabe, Yoshihiro Kawade, Masayoshi Hirohara

**Affiliations:** 1 Laboratory of Pharmacy Practice, Center for Education and Research on Clinical Pharmacy, Showa Pharmaceutical University, Machida, Tokyo, Japan; 2 Department of Medical Innovation, Nagoya City University Graduate School of Medicine, Nagoya, Aichi, Japan; 3 Center for Clinical Research, Nagoya City University West Medical Center, Nagoya, Aichi, Japan; 4 Department of Community-Based Medicine, Nagoya City University Graduate School of Medicine, Nagoya, Aichi, Japan; Xiamen University - Malaysia Campus, MALAYSIA

## Abstract

**Background:**

Community pharmacists play an important role in reducing COVID-19-related secondary health problems. However, the knowledge, attitudes, and practices (KAP) regarding COVID-19 among pharmacists in Japan have not yet been elucidated.

**Methods:**

We conducted a web-based questionnaire survey among 1,137 pharmacists working in health support pharmacies (HSPs) in Japan. These pharmacists are responsible for providing health consultations to community residents. We assessed COVID-19-related KAP among pharmacists and compared the results for two age groups: ≤49 years and ≥50 years. We used multiple regression analysis to examine which factors influence KAP scores regarding COVID-19.

**Results:**

From among the 2,141 HSPs in Japan, a total of 1,137 pharmacists, each representing a different HSP, responded to the survey. The results indicated that since the beginning of the COVID-19 pandemic, pharmacists have been providing consultations about COVID-19 to local residents, covering topics such as “Effective infection prevention methods” (60.6%) and “What the COVID-19 pandemic would be” (48.8%). Importantly, 73.5% of the pharmacists felt they “did not have enough information about COVID-19.” The main information resources about COVID-19 were Internet (91.2%) and television (78.9%). Across all respondents, the mean knowledge score (4.17/10) was lower than the mean scores for attitudes (7.26/10) and practices (5.79/10). Multiple regression analysis showed that having enough information about COVID-19 was a factor strongly associated with total KAP scores (*p*<0.001; 95% confidence interval, −1.344 to −0.540).

**Conclusions:**

Pharmacists working in community pharmacies provide residents with information related to COVID-19. In this role as a health partner, these pharmacists need a way to strengthen and expand their knowledge, and moreover, their ability to support community residents. Learning more about the available academic and scientific information, as well as having access to accurate epidemiological information, can offer a means of reaching these goals.

## Introduction

Severe acute respiratory syndrome coronavirus 2 (SARS-CoV-2), the causative agent of coronavirus disease 2019 (COVID-19), was first identified in Wuhan, Hubei Province, China [1, 2]. The virus rapidly spread around the world, and as of 28 June 2021, over 180 million cases and over 3.91 million deaths had been reported in countries all over the world [3].

Although most patients with COVID-19 are asymptomatic or only have mild illness [4], a few progress to acute respiratory illness and hypoxia, thus requiring hospitalization, and a subset of patients develop acute respiratory distress syndrome and/or have fatal outcomes [5, 6]. Public health measures implemented in response to COVID-19 have included self-isolation, social distancing, and shutting down all but essential services and industries. These abrupt changes to the usual patterns of human activity have had indirect negative effects on people’s physical and mental functioning [7]. Especially for older adults, staying at home continuously for a long time has caused reduced physical activity, changes in dietary intake, diminished muscle mass and physical functions, and accelerated frailty [8, 9]. Social isolation owing to COVID-19 has also had unintended adverse effects on older adults’ mental health, including the development of distress, anxiety, and depression [7, 8]. Particularly in Japan, where the population is aging, community health professionals have an important role in supporting older community-dwelling adults to forestall secondary health problems caused by COVID-19.

Community pharmacists are often the first health professionals from whom individuals receive information about COVID-19-related symptoms and with whom they speak about other COVID-19-related issues [10]. One report emphasizes the importance of public health education about COVID-19 that is offered by community pharmacists to local residents as well as the advice these pharmacists can provide if infection is suspected [11]. There are many problems associated with COVID-19, from infections to secondary health effects such as frailty. In view of the essential role of community pharmacists during the COVID-19 pandemic, the International Pharmaceutical Federation (FIP) published the FIP COVID-19 Guidance for Pharmacies and Pharmacists in response to the spread of this disease [12].

A health support pharmacy (HSP) is a specially registered community pharmacy within the Japanese health care system that fulfills the requirements of providing 24-hour consultation with a pharmacist and home care support to improve local residents’ quality of life [13, 14]. A knowledge, attitudes, and practices (KAP) survey is widely accepted means of assessing a person’s understanding of a given topic, their thinking in relation to that topic, and their associated skills [15]. Several KAP surveys related to COVID-19 have been conducted among community pharmacists in several countries [16–18]. The results of these surveys indicate that many pharmacists have adequate knowledge and perceptions as well as positive attitudes regarding COVID-19 [16–18]. Although pharmacists working in an HSP may also have adequate knowledge and positive attitudes related to COVID-19 and are very likely to assume the role of health partner with local residents to assist them with COVID-19-related issues, no KAP data regarding COVID-19 have been collected or evaluated among community pharmacists working in HSPs in Japan [19].

The purpose of the present study was to conduct a nationwide KAP survey among pharmacists working in HSPs to assess whether community pharmacists can contribute to reducing infections and the indirect secondary harm caused by COVID-19, and whether community pharmacists can potentially form beneficial health partnerships with local residents.

## Materials and methods

### Study design, site, and participants

We conducted a cross-sectional study using a web-based questionnaire survey over a 1-month period from October 1 to October 31, 2020. The inclusion criteria for this study were pharmacists who worked as the manager of an HSP in Japan (47 prefectures) and who agreed to participate. The exclusion criteria were those who did not agree to complete the survey and general pharmacists who were not a pharmacy manager.

The HSP was introduced into the Japanese health care system in 2016 by the Ministry of Health, Labour, and Welfare. At the time of the present study, 2,141 community pharmacies were registered as HSPs in Japan [13]. We invited pharmacists to participate via an announcement mailed to pharmacies nationwide. In the invitation package, we included a consent form and a self-addressed envelope. In this way, pharmacy managers who agreed to respond signed the consent form and returned it to us in the envelope. We provided the information for accessing the online survey only to those pharmacists who agreed to participate in this survey. The survey platform was Questant (Macromill, Inc., Minato-ku, Tokyo), which restricts survey access to one person using a unique internet protocol address. The web-based survey system used in this study could not move to the next page without answering the questions. Therefore, we collected questionnaires without any missing responses.

This study was approved by the Ethical Review Committee of Showa Pharmaceutical University (Reception/Approval No. 2020–5). Written informed consent was obtained from the study participants.

### Definitions of terms

With amendment of the School of Education Law-Japan in 2006, pharmacy schools are classified under two educational systems, a 4-year system and a 6-year system, and clinical education has been strengthened in the 6-year system. Subsequent to this amendment, only those who were educated at a pharmacy school under the 6-year system can now take the national pharmacist examination. People educated in the 4-year pharmacy school are no longer eligible to take the examination. Therefore, there were two types of pharmacist among participants in the present study, those who graduated from a 4-year pharmacy school before the law’s amendment and those who graduated from a 6-year pharmacy school.

### KAP survey

The KAP survey was conducted during the month of October 2020 using a self-administered web-based questionnaire. The questionnaire was developed by the study investigators according to the World Health Organization (WHO) Risk Communication and Community Engagement (RCCE) [20], “A guide to developing knowledge, attitude, and practice surveys” [15] as well as previous related studies [21–24] (S1 Appendix). The questionnaire was designed to assess KAP related to COVID-19 and to collect information about the following topics: participants’ demographic data, COVID-19-related information resources, pharmacy-based consultations, matters in which pharmacists felt they lacked knowledge, and information about COVID-19 that pharmacists felt was needed but they could not obtain, among other topics. All questions were either multiple choice or closed-ended questions. The response options for questions scored using a 3-point Likert scale were: “agree” (3 points), which represented a positive attitude; “neither agree/disagree” (2 points); and “disagree” (1 point), which represented a negative attitude. Scores for knowledge, attitudes, and practices were each calculated from the questionnaire responses. In the case of multiple choice, 1 point was assigned for each correct choice. For knowledge-related responses, cutoff points were set according to the number of correct answers, with 0 to 3 points assigned according to the number of correct answers [23, 24]. Because the number of questions and the total KAP score for each category (knowledge, attitudes, and practices) varied from item to item, the final score was recalculated such that the total possible score for each item was 10.

A pilot survey using the developed questionnaire was conducted among pharmacists who worked in settings other than community pharmacies, such as in hospitals or for academic organizations. The Cronbach’s alpha coefficient for internal consistency in the KAP score was 0.760.

Participants’ KAP results were summarized and then separated for comparison on the basis of pharmacies in high-risk versus those in low-risk areas. The number of COVID-19 cases at the time of the highest peak during the second wave of the pandemic in Japan (between June and September 2020) determined whether an area was high risk or low risk. Among the 47 examined prefectures across the country, 10 prefectures were classified as high-risk areas and included 95.8% of all COVID-19 cases at the time; these comprised Tokyo, Hokkaido, Kanagawa, Osaka, Saitama, Fukuoka, Aichi, Chiba, Kyoto, and Hyogo. All other prefectures were defined as low-risk areas.

### Statistical analysis

The collected data were expressed as mean and standard deviation (SD) or as median and interquartile range (IQR; 25%–75%) for continuous variables. Participating pharmacists were divided into two groups: those aged 49 years or younger and those aged 50 years or older. Comparisons between the two groups were made using the Mann–Whitney *U* test or Student *t*-test for continuous variables, and the χ^2^ test or Fisher’s exact test for categorical variables. To determine possible factors influencing KAP scores associated with COVID-19, demographic data (i.e., age, sex, years of experience as a pharmacist, pharmacy in a high-risk area, having enough information about COVID-19, and providing consultations to local residents) were chosen using a forced-entry method to conduct multiple regression analysis.

For all analyses, significance levels were two-tailed, and *p*<0.05 was set to indicate statistical significance. The analyses were carried out using IBM SPSS, version 27.0 (IBM Corp., Armonk, NY, USA).

## Results

### Demographic data of pharmacists working in health support pharmacies

Of the 2,141 recruited pharmacists who were managers of an HSP, 1,137 (53.1%) agreed to participate in the study and completed the web-based questionnaire. The demographic data of participants are shown in Table 1.

**Table 1:** Demographic data of HSP pharmacists.

	Total	Age ≤49 years	Age ≥50 years (N = 377)	*p* value
	N = 1,137	(N = 760)		
**Age, median (IQR)**	44 (35–53)	38 (34–44)	57 (53–62)	<0.001
**Sex, n (%)**				<0.001
Male	626 (55.1)	480 (63.2)	146 (38.7)	
Female	511 (44.9)	280 (36.8)	231 (61.3)	
**Academic background, n (%)**				<0.001
4-year university	844 (74.2)	497 (65.4)	347 (92.0)	
6-year university	192 (16.9)	186 (24.5)	6 (1.6)	
Master’s degree	87 (7.7)	65 (8.6)	22 (5.8)	
Doctoral degree	14 (1.2)	12 (1.6)	2 (0.5)	
**Years of experience as a pharmacist, median (IQR)**	17 (10–25)	13 (9–19)	28 (22–35)	<0.001
**Number of pharmacies^a^**				<0.001
1 to 5 pharmacies	425 (37.4)	197 (25.9)	228 (60.5)	
6 to 50 pharmacies	262 (23.0)	196 (25.8)	66 (17.5)	
>50 pharmacies	450 (39.6)	367 (48.3)	83 (22.0)	
**Number of pharmacist coworkers, n (%) (N = 1,092)**				0.006
2 to 3	332 (30.4)	211 (28.8)	121 (33.6)	
4 to 5	360 (33.0)	229 (31.3)	131 (36.4)	
≥6	400 (36.6)	292 (39.9)	108 (30.0)	
**Pharmacy in high-risk area^b^, n (%)**	563 (49.5)	384 (50.5)	179 (47.5)	0.333
**Number of people living in the same household, n (%)**				<0.001
Living alone	171 (15.0)	134 (17.6)	37 (9.8)	
Living with a spouse	231 (20.3)	99 (13.0)	132 (35.0)	
Living with spouse and children or grandchildren	494 (43.4)	369 (48.6)	125 (33.2)	
No spouse, living with children and grandchildren	32 (2.8)	16 (2.1)	16 (4.2)	
Living with parents and grandparents	107 (9.4)	80 (10.5)	27 (7.2)	
Living with three generations	53 (4.7)	36 (4.7)	17 (4.5)	
Other	49 (4.3)	26 (3.4)	23 (6.1)	
**Number of family members, n (%)**				<0.001
Living alone	184 (16.2)	137 (18.0)	47 (12.5)	
2 people	266 (23.4)	124 (16.3)	142 (37.7)	
3 people	277 (24.4)	184 (24.2)	93 (24.7)	
4 people	273 (24.0)	212 (27.9)	61 (16.2)	
≥5 people	137 (12.0)	103 (13.6)	34 (9.0)	
**Caring for a family member, n (%)**	74 (6.5)	21 (2.8)	53 (14.1)	<0.001
**Underlying disease in pharmacist, n (%)**				
None	899 (79.1)	679 (89.3)	220 (58.4)	<0.001
Cerebral infarction	6 (0.5)	2 (0.3)	4 (1.1)	0.098
Hypertension	117 (10.3)	25 (3.3)	92 (24.4)	<0.001
Angina / Arrhythmia	12 (1.1)	4 (0.5)	8 (2.1)	0.018
Diabetes	27 (2.4)	5 (0.7)	22 (5.8)	<0.001
COPD / Asthma	22 (1.9)	7 (0.9)	15 (4.0)	<0.001
Chronic renal disease	6 (0.5)	5 (0.7)	1 (0.3)	0.354
Chronic liver disease	6 (0.5)	4 (0.5)	2 (0.5)	0.646
Other	94 (8.3)	38 (5.0)	56 (14.9)	<0.001

Abbreviations: HSP, health support pharmacy; IQR, interquartile range; COPD, chronic obstructive pulmonary disease.

^a^ Number of pharmacies owned by the same company.

^b^ Prefectures in Japan that were high-risk areas for COVID-19 infection during the second wave of the pandemic.

The median age of participating pharmacists was 44 years, with 66.8% age 49 years or younger. Men constituted 55.1% of all participants, and 74.2% of all participants had a 4-year university education. Most respondents stated that the number of pharmacies owned by the company for which they worked was more than 50 pharmacies (39.6%), and most of these employed more than six pharmacists per store (36.6%). The median number of years of experience working as a pharmacist was 17 years; 79.1% of pharmacists had no underlying diseases.

### Consultations with local residents about COVID-19

**Fig 1:**
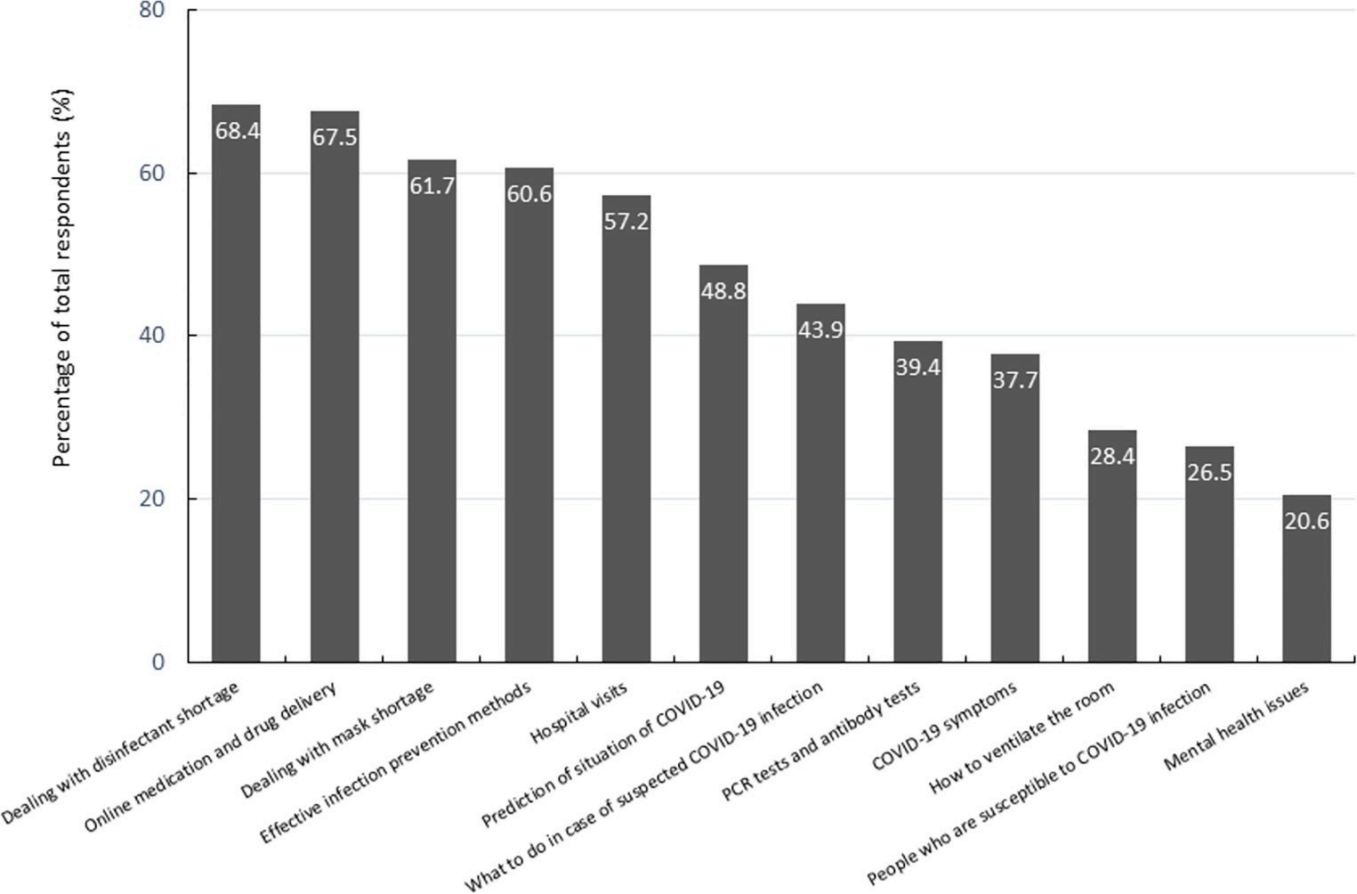
COVID-19-related topics discussed in pharmacists’ consultations with local residents. Multiple-response data were obtained on COVID-19-related consultations provided to local residents by pharmacists in health support pharmacies.

Fig 1 shows the most common topics discussed during consultations related to COVID-19 provided to local residents by pharmacists during the COVID-19 pandemic.

The most common topics covered during consultations with pharmacists were dealing with disinfectant shortages (68.4%), online medication and drug delivery (67.5%) and other medication-related questions, and dealing with mask shortages (61.7%), followed by effective infection prevention methods and mental health.

Pharmacists working in low-risk areas had significantly more consultations regarding effective infection prevention methods than those in high-risk areas whereas pharmacists in high-risk areas had more consultations regarding what to do in case of a suspected COVID-19 infection and PCR tests and antibody tests (S1 Fig).

### Information resources related to COVID-19 among pharmacists working in health support pharmacies

**Fig 2:**
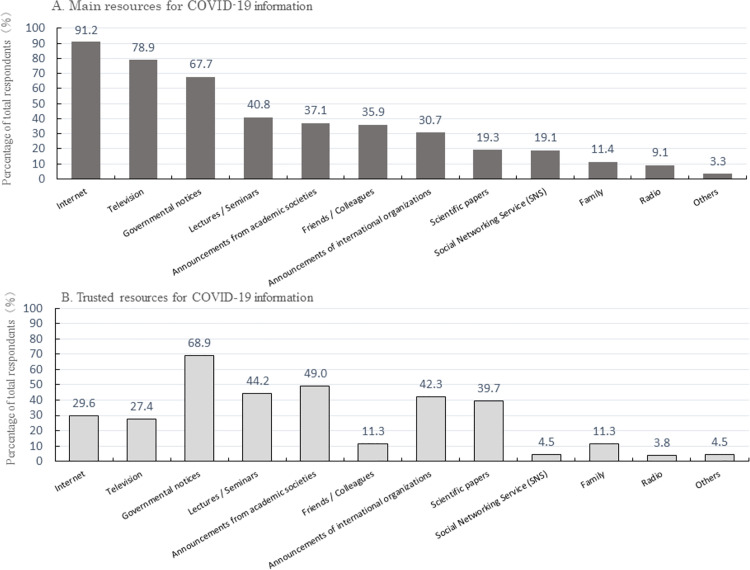
(a) Main resources for COVID-19 information; (b) trusted resources for COVID-19 information.

Fig 2 presents the main and trusted COVID-19 information resources on which participants relied.

The most common COVID-19-related information resource was the Internet (91.2%), followed by television (78.9%) and governmental notices (67.7%). Scientific papers (19.3%), announcements from academic societies (37.1%) and announcements from international organizations (30.7%) were less frequently reported as a main source of information on COVID-19.

Fig 2B shows the COVID-19-related information resources trusted by participants. Governmental notices was the most trusted resource (68.9%), followed by announcements from academic societies (49.0%), lectures and seminars (44.2%), and announcements from international organizations (42.3%). Slightly under 30% of respondents trusted television (27.4%) and the Internet (29.6%).

In a comparison between pharmacists working in high- and low-risk areas, a significantly higher proportion in low-risk areas trusted information received in lectures and seminars, and a significantly higher proportion in high-risk areas trusted information received from friends and colleagues (S2 Fig).

### Knowledge related to COVID-19 among pharmacists working in health support pharmacies

The results of questions related to knowledge about COVID-19 are shown in Table 2.

**Table 2:** Pharmacists’ responses regarding COVID-19-related knowledge, by age group.

	Total	Age ≤49 years (N = 760)	Age ≥50 years (N = 377)	*p* value
	N = 1,137			
**Knowledge score, mean (SD)**	4.17 (1.24)	4.07 (1.24)	4.36 (1.23)	<0.001
**I have enough information about COVID-19, n (%)**				0.001
Yes	174 (15.3)	100 (13.2)	74 (19.6)	
No	836 (73.5)	561 (73.8)	275 (72.9)	
Do not know	127 (11.2)	99 (13.0)	28 (7.4)	
**COVID-19-related information that is needed but not available, n (%)**				
Therapeutic drugs and vaccines	694 (61.0)	457 (60.1)	237 (62.9)	0.374
Infection prevention	240 (21.1)	160 (21.1)	80 (21.2)	0.948
Post-infection pathology	525 (46.2)	350 (46.1)	175 (46.4)	0.907
Test information such as PCR and antibody tests	626 (55.1)	399 (52.5)	227 (60.2)	0.014
Mental health care for patients and medical staff	349 (30.7)	253 (33.3)	96 (25.5)	0.007
Specific measures to be taken when pharmacy staff are infected	517 (45.5)	354 (46.6)	163 (43.2)	0.287
Where to find trustworthy information resources	545 (47.9)	364 (47.9)	181 (48.0)	0.971
Other	22 (1.9)	14 (1.8)	8 (2.1)	0.747
**Information that will be needed in the future to coexist with COVID-19, n (%)**				
Accurate epidemiological data	871 (76.6)	592 (77.9)	279 (74.0)	0.145
Predicting the spread or resolution of infections	493 (43.4)	338 (44.5)	155 (41.1)	0.282
Infected areas	413 (36.3)	286 (37.6)	127 (33.7)	0.193
Contact information in case of suspected infection	659 (58.0)	435 (57.2)	224 (59.4)	0.483
Where to get a PCR test	746 (65.6)	488 (64.2)	258 (68.4)	0.158
Countries where overseas travel is possible	180 (15.8)	125 (16.4)	55 (14.6)	0.419
Websites that can collect both medical and pharmaceutical information	542 (47.7)	349 (45.9)	193 (51.2)	0.094
Materials and handouts for use in pharmacies	642 (56.5)	413 (54.3)	229 (60.7)	0.040
Mental health measures	387 (34.0)	269 (35.4)	118 (31.3)	0.170
Other	14 (1.2)	12 (1.6)	2 (0.5)	0.106
**What I know about COVID-19, n (%)**				
I do not know anything about COVID-19	18 (1.6)	12 (1.6)	6 (1.6)	0.987
Required infection protection measures	773 (68.0)	487 (64.1)	286 (75.9)	<0.001
Symptoms of infected people	813 (71.5)	545 (71.7)	268 (71.1)	0.827
Routes of infection	514 (45.2)	363 (47.8)	151 (40.1)	0.014
Actions to be taken at the onset of infection	835 (73.4)	542 (71.3)	293 (77.7)	0.021
I know that there are asymptomatic cases of COVID-19	1004 (88.3)	665 (87.5)	339 (89.9)	0.232
Risks of symptom aggravation	882 (77.6)	579 (76.2)	303 (80.4)	0.111
Required medical care system for COVID-19 patients	296 (26.0)	190 (25.0)	106 (28.1)	0.260
Mortality risk	406 (35.7)	269 (35.4)	137 (36.3)	0.754
Consultation counter for COVID-19	422 (37.1)	268 (35.3)	154 (40.8)	0.066
**People at high risk of COVID-19 infection (correct responses), n (%)**	229 (20.1)	137 (18.0)	92 (24.4)	0.012
**How COVID-19 is spread (correct responses), n (%)**	37 (3.3)	23 (3.0)	14 (3.7)	0.539
**Main symptoms of COVID-19 (correct responses), n (%)**	238 (20.9)	139 (18.3)	99 (26.3)	0.002
**What is the right way to prevent COVID-19? (correct responses), n (%)**	55 (4.8)	43 (5.7)	12 (3.2)	0.067
**I think I might be infected with COVID-19, n (%)**	1110 (97.6)	748 (98.4)	362 (96.0)	0.012
**No antiviral drugs are effective against COVID-19, n (%)**				<0.001
Yes	659 (58.0)	412 (54.2)	247 (65.5)	
No	182 (16.0)	121 (15.9)	61 (16.2)	
Do not know	296 (26.0)	227 (29.9)	69 (18.3)	

The mean (SD) knowledge score for questions related to COVID-19 was 4.17 (1.24) out of 10 points. Older pharmacists had a significantly higher knowledge score than their younger counterparts; however, 73.5% of all participants felt they did not have sufficient information about COVID-19. Most reported that necessary but unavailable information was related to therapeutic drugs and vaccines (61.0%), and the most frequently reported information that was needed to be able to coexist with COVID-19 was accurate epidemiological information (76.6%).

Significantly more older pharmacists than younger pharmacists said they knew about required infection protection measures (75.9%) against COVID-19 and actions to be taken at the onset of infection (77.7%). Younger pharmacists were significantly more likely than older pharmacists to know the routes of infection (47.8%). However, for questions directly related to COVID-19 (i.e., identifying high-risk individuals, routes of infection, main COVID-19 symptoms, and correct preventive measures), the percentage of accurate responses was low in both groups.

### Attitudes related to COVID-19 among pharmacists working in health support pharmacies

The results of pharmacists’ responses to questions addressing COVID-19-related attitudes are shown in Table 3.

**Table 3:** Pharmacists’ responses regarding attitudes related to COVID-19.

	Total	Age ≤49 years (N = 760)	Age ≥50 years (N = 377)	*p* value
	N = 1,137			
**Attitudes score, mean (SD)**	7.26 (1.54)	7.18 (1.56)	7.41 (1.51)	0.015
**My job puts me at high risk for COVID-19 infection, n (%)**				0.190
Agree	837 (73.6)	556 (73.2)	281 (74.5)	
Disagree	191 (16.8)	123 (16.2)	68 (18.0)	
Neither agree/disagree	109 (9.6)	81 (10.7)	28 (7.4)	
**How much of a threat is COVID-19? n (%)**				0.761
A serious threat	293 (25.8)	201 (26.4)	92 (24.4)	
A neutral threat	669 (58.8)	440 (57.9)	229 (60.7)	
Not a threat	165 (14.5)	113 (14.9)	52 (13.8)	
Other	10 (0.9)	6 (0.8)	4 (1.1)	
**I will feel embarrassed if I become infected with COVID-19, n (%)**	263 (23.1)	176 (23.2)	87 (23.1)	0.976
**I think that the COVID-19 situation will be resolved in the near future, n (%)**				0.383
Agree	555 (48.8)	360 (47.4)	195 (51.7)	
Disagree	283 (24.9)	195 (25.7)	88 (23.3)	
Neither agree/disagree	299 (26.3)	205 (27.0)	94 (24.9)	
**The infection control measures in the pharmacy where I work are sufficient, n (%)**				0.002
Agree	614 (54.0)	385 (50.7)	229 (60.7)	
Disagree	197 (17.3)	134 (17.6)	63 (16.7)	
Neither agree/disagree	326 (28.7)	241 (31.7)	85 (22.5)	
**I want to keep my current job despite the high risk of COVID-19 infection, n (%)**	1101 (96.8)	732 (96.3)	369 (97.9)	0.157
**I am proud of my job, n (%)**	1087 (95.6)	719 (94.6)	368 (97.6)	0.020
**COVID-19 is a preventable disease, n (%)**				0.669
Agree	670 (58.9)	441 (58.0)	229 (60.7)	
Disagree	133 (11.7)	90 (11.8)	43 (11.4)	
Neither agree/disagree	334 (29.4)	229 (30.1)	105 (27.9)	
**Influenza is a preventable disease, n (%)**				0.519
Agree	908 (79.9)	601 (79.1)	307 (81.4)	
Disagree	132 (11.6)	94 (12.4)	38 (10.1)	
Neither agree/disagree	97 (8.5)	65 (8.6)	32 (8.5)	
**Factors that make COVID-19 feel threatening, n (%)**				
It does not feel threatening	29 (2.6)	22 (2.9)	7 (1.9)	0.296
I might become infected	803 (70.6)	516 (67.9)	287 (76.1)	0.004
I might infect others	935 (82.2)	639 (84.1)	296 (78.5)	0.021
I might die	289 (25.4)	202 (26.6)	87 (23.1)	0.202
If I get infected, I might be quarantined	354 (31.1)	237 (31.2)	117 (31.0)	0.959
My work and daily life will be restricted	909 (79.9)	600 (78.9)	309 (82.0)	0.232
There is no effective medicine or vaccine	619 (54.4)	394 (51.8)	225 (59.7)	0.012
Other	54 (4.7)	39 (5.1)	15 (4.0)	0.390
Do not know	4 (0.4)	3 (0.4)	1 (0.3)	0.596
**What do you worry about if you became infected with COVID-19? n (%)**				
Prejudice and discrimination in my neighborhood and workplace	667 (58.7)	466 (61.3)	201 (53.3)	0.010
Causing trouble in the workplace	1082 (95.2)	723 (95.1)	359 (95.2)	0.945
Family will be subjected to prejudice or discrimination in school or the workplace	602 (52.9)	436 (57.4)	166 (44.0)	<0.001
I may not heal (I will die)	189 (16.6)	118 (15.5)	71 (18.8)	0.159
Passing the infection on to someone else	1000 (88.0)	661 (87.0)	339 (89.9)	0.151
Causing outbreaks of infectious disease	786 (69.1)	528 (69.5)	258 (68.4)	0.721
Decreased income owing to leave of absence	352 (31.0)	244 (32.1)	108 (28.6)	0.235
Payment for medical treatment	171 (15.0)	127 (16.7)	44 (11.7)	0.025
Having no one to entrust my work to while recuperating	416 (36.6)	257 (33.8)	159 (42.2)	0.006
Having no one to delegate household chores to while recuperating	128 (11.3)	75 (9.9)	53 (14.1)	0.035
Having no one to entrust the long-term care of my family to while recuperating	54 (4.7)	25 (3.3)	29 (7.7)	0.001
Other	17 (1.5)	14 (1.8)	3 (0.8)	0.171
**Things that have influenced your own precautionary behavior, n (%)**				
Government state of emergency	1,033 (90.9)	690 (90.8)	343 (91.0)	0.916
WHO pandemic declaration	369 (32.5)	224 (29.5)	145 (38.5)	0.002
Announcement of postponement of the Tokyo Olympics	139 (12.2)	92 (12.1)	47 (12.5)	0.861
Celebrity deaths from COVID-19 infection	396 (34.8)	254 (33.4)	142 (37.7)	0.157
COVID-19 infection in people close to me	44 (3.9)	28 (3.7)	16 (4.2)	0.645
Death of people close to me from COVID-19 infection	13 (1.1)	6 (0.8)	7 (1.9)	0.100
COVID-19 infection in celebrities	199 (17.5)	121 (15.9)	78 (20.7)	0.046
Other	87 (7.7)	64 (8.4)	23 (6.1)	0.166
**Concerns at working at a pharmacy because of the spread of COVID-19 infection, n (%)**				
Risk of being infected	916 (80.6)	610 (80.3)	306 (81.2)	0.717
Securing masks and disinfectants	432 (38.0)	303 (39.9)	129 (34.2)	0.065
Securing medicines	242 (21.3)	179 (23.6)	63 (16.7)	0.008
Maintaining operational staff	599 (52.7)	399 (52.5)	200 (53.1)	0.861
Workload owing to pharmaceutical deliveries	152 (13.4)	105 (13.8)	47 (12.5)	0.529
Decrease in visiting patients	729 (64.1)	507 (66.7)	222 (58.9)	0.010
Infection prevention measures in the pharmacy	633 (55.7)	426 (56.1)	207 (54.9)	0.714
Lack of information related to COVID-19	367 (32.3)	249 (32.8)	118 (31.3)	0.619
Workload from increase in long-term prescriptions	210 (18.5)	127 (16.7)	83 (22.0)	0.030
Other	10 (0.9)	9 (1.2)	1 (0.3)	0.105

Abbreviation: SD, standard deviation.

The mean (SD) attitude score was 7.26 (1.54) out of 10 points. Older pharmacists had a significantly higher attitude score related to COVID-19 than their younger counterparts. Of the total, 95.6% of pharmacists reported that they were proud of their job and 73.6% felt that their job put them at high risk for COVID-19 infection. However, despite having a high-risk job, 96.8% of pharmacists said they wished to remain in their current job. The government’s state of emergency declaration was the single most (90.9%) influential factor that affected pharmacists’ precautionary behaviors. The WHO pandemic declaration was mentioned by 32.5% of all pharmacists, with significantly more older pharmacists (38.5%) than younger (29.5%) pharmacists influenced by the declaration (*p* = 0.002). Among all participants, 23.1% said they would feel embarrassed if they were infected with COVID-19; the top concerns associated with contracting with COVID-19 infection were the fear of causing problems at work (95.2%) and fear of infecting someone else (88.0%). Younger pharmacists (57.4%) were significantly more likely than older pharmacists (44.0%) to worry about prejudice and discrimination against themselves and their families as a result of infection (*p*<0.001). Furthermore, 84.6% of all respondents felt threatened by COVID-19; the reasons most commonly reported were fear of passing the infection on to someone else (82.2%) and concerns that their work and daily life would be restricted (79.9%). In a comparison of responses by pharmacists in the two age groups regarding attitudes about the threat of COVID-19, older pharmacists were significantly more likely to report fear of contracting the infection themselves (76.1%) and younger pharmacists were significantly more likely to fear infecting someone else (84.1%).

Regarding the pharmacies where respondents worked, 54% of pharmacists said that there were adequate infection control measures in place. Significantly more pharmacists in the older group felt the infection control measures at their pharmacy were sufficient (60.7%). However, the risk of contracting COVID-19 infection (80.6%) was the number one concern when working at the pharmacy. Younger pharmacists were significantly more likely than older pharmacists to be concerned about the decline in the number of pharmacy customers and the availability of medicines. Among all respondents, 48.8% reported that they felt the COVID-19 pandemic would end soon; 58.9% believed that COVID-19 is a preventable disease, and 79.9% said that influenza is preventable.

### Practices related to COVID-19

The results regarding questions about COVID-19-related practices are shown in Table 4.

**Table 4:** Pharmacists’ responses regarding practices related to COVID-19.

	Total	Age ≤49 years (N = 760)	Age ≥50 years (N = 377)	*p* value
	N = 1,137			
**Practices score, mean (SD)**	5.79 (0.97)	5.80 (0.96)	5.77 (0.98)	0.531
**What to watch for in clients suspected of having COVID-19 infection in the home, n (%)**				
Separate rooms for infected people	1,036 (91.1)	690 (90.8)	346 (91.8)	0.582
Limit the number of people caring for the infected person	916 (80.6)	611 (80.4)	305 (80.9)	0.839
Make everyone wear a mask	989 (87.0)	653 (85.9)	336 (89.1)	0.131
Gargle and wash hands frequently	1,051 (92.4)	697 (91.7)	354 (93.9)	0.189
Ventilate as much as possible	982 (86.4)	657 (86.4)	325 (86.2)	0.911
Disinfect common areas	980 (86.2)	644 (84.7)	336 (89.1)	0.043
Wash soiled linens and clothes	661 (58.1)	435 (57.2)	226 (59.9)	0.383
Dispose of trash in a sealed container	755 (66.4)	482 (63.4)	273 (72.4)	0.003
Other	44 (3.9)	28 (3.7)	16 (4.2)	0.645
**What to do if you or a family member has symptoms that suggest COVID-19 infection, n (%)**				
Seek medical attention immediately	281 (24.7)	178 (23.4)	103 (27.3)	0.151
Call the health center for advice	936 (82.3)	637 (83.8)	299 (79.3)	0.061
Contact the workplace	878 (77.2)	603 (79.3)	275 (72.9)	0.015
Talk to family	480 (42.2)	318 (41.8)	162 (43.0)	0.717
Talk to a friend or colleague	129 (11.3)	89 (11.7)	40 (10.6)	0.582
Consult the family doctor	382 (33.6)	194 (25.5)	188 (49.9)	<0.001
Go to a pharmacy or drug store to buy medicine	5 (0.4)	3 (0.4)	2 (0.5)	0.536
Go to the pharmacy and consult a pharmacist	9 (0.8)	3 (0.4)	6 (1.6)	0.041
Do not tell anyone	11 (1.0)	8 (1.1)	3 (0.8)	0.477
Do not go out of the house	467 (41.1)	313 (41.2)	154 (40.8)	0.914
Other	23 (2.0)	17 (2.2)	6 (1.6)	0.467
**Infection measures I have in place for COVID-19, n (%)**				
Wear a mask where there are other people	1,123 (98.8)	748 (98.4)	375 (99.5)	0.106
Wash hands regularly using alcohol-based hand sanitizer or soap and water	1,122 (98.7)	748 (98.4)	374 (99.2)	0.212
Gargle	838 (73.7)	569 (74.9)	269 (71.4)	0.205
Ventilate the room regularly	1,000 (88.0)	659 (86.7)	341 (90.5)	0.068
Room cleaning and disinfection	638 (56.1)	421 (55.4)	217 (57.6)	0.489
Refrain from going out on holidays	600 (52.8)	386 (50.8)	214 (56.8)	0.057
Avoid contact with people as much as possible	623 (54.8)	431 (56.7)	192 (50.9)	0.065
Maintain a nutritionally balanced diet	670 (58.9)	415 (54.6)	255 (67.6)	<0.001
Get enough sleep	718 (63.1)	454 (59.7)	264 (70.0)	0.001
I do not do anything	2 (0.2)	1 (0.1)	1 (0.3)	0.553
Other	13 (1.1)	7 (0.9)	6 (1.6)	0.236
**Infection control measures implemented in the pharmacy, n (%)**				
Disinfect indoor items and equipment	1,008 (88.7)	677 (89.1)	331 (87.8)	0.522
Ventilate the room regularly	1,086 (95.5)	720 (94.7)	366 (97.1)	0.072
Alert and educate patients about COVID-19	768 (67.5)	497 (65.4)	271 (71.9)	0.028
Limit the number of patients allowed into the pharmacy	216 (19.0)	142 (18.7)	74 (19.6)	0.702
Thorough hand washing and masks for pharmacy staff	1,108 (97.4)	738 (97.1)	370 (98.1)	0.296
Remove common items such as magazines and books	782 (68.8)	550 (72.4)	232 (61.5)	<0.001
Encourage cashless payment	376 (33.1)	253 (33.3)	123 (32.6)	0.823
Offer recommendations for how to request medications online	158 (13.9)	105 (13.8)	53 (14.1)	0.911
Install shields to protect against infection	1,015 (89.3)	673 (88.6)	342 (90.7)	0.267
Other	23 (2.0)	14 (1.8)	9 (2.4)	0.539
I do not do anything	1 (0.1)	0 (0)	1 (0.3)	0.332
**I get the flu vaccine every year, n (%)**				0.003
Yes	969 (85.2)	656 (86.3)	313 (83.0)	
No	96 (8.4)	50 (6.6)	46 (12.2)	
Sometimes	72 (6.3)	54 (7.1)	18 (4.8)	
**Were you in the habit of wearing a mask even before the COVID-19 pandemic? n (%)**				0.012
Yes	58 (5.1)	36 (4.7)	22 (5.8)	
Only in the cold and flu season	752 (66.1)	525 (69.1)	227 (60.2)	
No	327 (28.8)	199 (26.2)	128 (34.0)	
**Were hand washing and hand hygiene practices in place before the COVID-19 pandemic? n (%)**				0.074
Yes	791 (69.6)	545 (71.7)	246 (65.3)	
Only in the cold and flu season	287 (25.2)	180 (23.7)	107 (28.4)	
No	59 (5.2)	35 (4.6)	24 (6.4)	
**What I was doing to prevent the flu, n (%)**				
Wore a mask around other people	887 (78.0)	608 (80.0)	279 (74.0)	0.022
Washed my hands and gargled after going out	1,058 (93.1)	709 (93.3)	349 (92.6)	0.655
Followed good coughing etiquette	931 (81.9)	614 (80.8)	317 (84.1)	0.174
Avoided places where people gather as much as possible	358 (31.5)	237 (31.2)	121 (32.1)	0.755
Refrained from business trips and travel	99 (8.7)	51 (6.7)	48 (12.7)	0.001
Received influenza vaccination	902 (79.3)	612 (80.5)	290 (76.9)	0.158
Other	12 (1.1)	8 (1.1)	4 (1.1)	0.603
**I have participated in academic society events, workshops, and study sessions on COVID-19, n (%)**	351 (30.9)	204 (26.8)	147 (39.0)	<0.001
**I am involved in the treatment and care of patients with COVID-19, n (%)**	102 (9.0)	64 (8.4)	38 (10.1)	0.357

Abbreviation: SD, standard deviation.

The mean (SD) overall practices score was 5.79 (0.97) out of 10. There were no significant differences in COVID-19-related practice scores between the two age groups. In terms of advice given to pharmacy customers suspected of having COVID-19 infection when they were at home, a high percentage of respondents recommended basic infection control measures, with the top responses being to gargle and wash hands frequently (92.4%), keep infected people in separate rooms (91.1%), and make sure that everyone wore a mask (87.0%). The two most common responses to the question of what to do if they or their family have symptoms suggesting COVID-19 infection were to call the health center for advice (82.3%) and contact the workplace (77.2%).

As for personal COVID-19 infection control measures, nearly all respondents reported that they wear a mask in public (98.8%) and wash their hands regularly using alcohol-based hand sanitizer or soap and water (98.7%). The most common infection control measures taken at pharmacies were thorough hand washing and use of masks by pharmacy staff (97.4%), followed by ventilating the room regularly (95.5%).

A high percentage (85.2%) of respondents said they were vaccinated against influenza every year, 69.6% habitually wore masks prior to the COVID-19 pandemic, and 69.6% had practiced hand washing and hand hygiene before the pandemic. The top two things pharmacists did to prevent influenza were to receive influenza vaccination (79.3%) and wear a mask in public places (78.0%).

Older pharmacists were significantly more likely than their younger counterparts to take precautions if a client was suspected of having COVID-19 infection and to implement control measures against infection. The older group was also significantly more likely than the younger group to have participated in academic society activities, workshops, and study sessions about COVID-19.

### Factors influencing scores for COVID-19-related knowledge, attitudes, and practices

We estimated the factors influencing each individual score and total KAP scores associated with COVID-19 using a multiple regression model (Table 5).

**Table 5:** Factors influencing individual and total KAP scores associated with COVID-19 in multiple regression analysis.

	Knowledge score		Attitudes score		Practices score		KAP total score	
	*p* value	95% CI	*p* value	95% CI	*p* value	95% CI	*p* value	95% CI
Age	0.732	−0.015–0.010	0.088	−0.002–0.029	0.350	−0.015–0.005	0.597	−0.018–0.032
Sex	0.067	−0.010–0.283	0.301	−0.282–0.087	0.001	0.082–0.314	0.117	−0.060–0.534
Having enough information about COVID-19	<0.001	−0.718 to −0.321	0.012	−0.572 to −0.072	0.212	−0.256–0.057	<0.001	−1.344 to −0.540
Pharmacy in a high-risk area	0.814	−0.160–0.125	0.432	−0.252–0.108	0.672	−0.137–0.088	0.441	−0.402–0.175
Years of experience as a pharmacist	0.021	0.002–0.030	0.474	−0.024–0.011	0.996	−0.011–0.011	0.488	−0.018–0.038
Being consulted by residents	0.320	−0.605–0.198	0.343	−0.751–0.261	0.281	−0.491–0.143	0.133	−1.435–0.190

Abbreviations: CI, confidence interval; KAP, knowledge, attitudes, and practices.

Baseline adjusted covariates: Age, sex, years of experience as a pharmacist, pharmacy in a high-risk area, having enough information about COVID-19, being consulted by residents.

Having enough information about COVID-19 and years of experience working as a pharmacist significantly affected the COVID-19 knowledge score. Having enough COVID-19 information significantly influenced the attitudes score, and sex significantly affected the practices score. Finally, having enough information about COVID-19 was the factor that most significantly affected the total KAP score.

## Discussion

The present study revealed that, throughout the COVID-19 pandemic, pharmacists working in community pharmacies in Japan have provided consultations to local residents about issues related to COVID-19 more than about medications related to usual issues. The most commonly accessed information resources regarding COVID-19 reported by pharmacists were television and the Internet; academic society notices and scientific papers were used to a lesser extent. Knowledge scores were quite low in both older and younger groups; however, those of pharmacists in the older age group were significantly higher than scores in the younger group. Not having sufficient information about COVID-19 was the factor most strongly related to the total KAP score among pharmacists working in community pharmacies. Our results suggested that, under conditions of the COVID-19 pandemic, community pharmacists can be effective health partners for local residents. Acquiring relevant scientific information and proper clinical and epidemiological information can arguably contribute to expanding and strengthening pharmacists’ ability to support local residents by providing well-informed information regarding COVID-19 prevention and control measures.

### Information resources on COVID-19 among pharmacists in health support pharmacies

A survey report on community pharmacists in Japan between 2016 and 2019 [25] showed that 70% of all consultation issues were regarding current symptoms, prescription medications, and regular medications. Indeed, medication issues are typically the predominant topic in consultations with community pharmacists. During periods of widespread COVID-19 infections, HSP pharmacists in Japan have consulted with many local residents on topics other than medications, including clinical and epidemiological information related to COVID-19, mental health issues, psychological problems such as social isolation, and a lack of sleep owing to taking excessive precautions to prevent infection. However, more than 70% of HSP pharmacists reported that they did not have sufficient information about COVID-19. Their main resources for COVID-19-related information were television and the Internet, which is no different from the resources (and information) available to the local population. A few pharmacists relied on scientific papers and announcements from academic societies and international organizations as their main information source. These choices may be reflected numerically in the knowledge scores among participants, especially those in the younger age group. Pharmacists working in low-risk areas received significantly more information in workshops and lectures whereas those working in high-risk areas relied significantly more on information from colleagues and friends, revealing a problem in the way information sources are made available. These results indicate that community pharmacists actively seek accurate information, even if they work in a pharmacy located in a low-risk area. However, the average knowledge score (4 out of 10 points) was lower than the scores for attitudes and practices. Previous reports on KAP surveys about COVID-19 among pharmacists in different countries have shown adequate knowledge scores among respondents [16–18]; however, these studies were limited to very basic information, making a direct comparison with our results difficult. To provide professional consultations that are better informed, with specialized knowledge and information, pharmacists must be able to obtain such information from scientific papers and official notifications related to COVID-19. In the present study, over 95% of pharmacists reported having pride in their job, with a higher prevalence among older pharmacists. Having additional scientific information would help fill knowledge gaps, thereby increasing community pharmacists’ confidence in sharing useful information when consulting with customers about COVID-19. Ultimately, these improvements will help pharmacists provide better health management for local residents.

### Role of community pharmacists in the control of COVID-19 spread

Previous reports from Canada, Japan, and the United Kingdom have raised concerns about the increase of physical, mental, and social frailty in older adults as a result of public health measures taken in response to the COVID-19 pandemic [7–9]. Japan is a super-aging society, and it is reported that 28.7% (2020) of the total population is aged 65 and over. Older adults living in the community must be cared for by health care professionals, at least in part. Pharmacists are not only readily available to local residents to provide health consultations but these health professionals can also provide disease management that incorporates safe drug treatment in older adults. HSP pharmacists in Japan contribute to the public health of the community by providing two main pharmacy services: primary care, including 24-hour pharmacy and home services, and health education programs for disease prevention and health promotion [13, 14]. In such an environment, pharmacists have the potential to simultaneously provide medication management and COVID-19 infection control for the population. In particular, given that chronic diseases such as hypertension and diabetes raise a person’s risk for developing severe COVID-19, by fulfilling their aforementioned dual role, pharmacists can help to reduce the risk of severe disease among local residents. It has been reported that during the pandemic, older people living in the community not only refrain from going outside of the home because of taking excessive precautions but they also refrain from going to medical clinics. Older age is a risk factor for COVID-19, and therefore older people in particular must have a place where they can obtain accurate and easy-to-understand information about COVID-19. Although the scope of services provided by community pharmacies varies by country, community pharmacies in many countries have a variety of public health roles in response to the COVID-19 pandemic. These pharmacies provide appropriate health care services to the local population such as ensuring a stable supply of medicines and hygiene products and providing screening and consultation recommendations for COVID-19, patient education, and psychological support [11, 26, 27]. According to the results of this study, there have been fewer medication-related consultations during the pandemic than previously [25]. However, community pharmacists must further strengthen consultations regarding drug therapy. From the perspective of the local population, community pharmacists are expected to undertake roles as both a manager of underlying diseases, to prevent the risk and severity of COVID-19 infection, and as an approachable scientist with extensive evidence-based expertise in drug therapy.

The most common response from HSP pharmacists to the question of what COVID-19-related information will be needed in the future was accurate epidemiological data (Table 2). A national survey of community pharmacists in Italy [27] showed that pharmacists’ science-based expertise was highly valued by their clients in the face of uncertain or false information about COVID-19 and related matters; however, no examples were given of pharmacies that also provide epidemiological information. Although numerous epidemiological studies on COVID-19 have been conducted in Japan, few implementation studies have been carried out that can link university researchers with community pharmacies and provide feedback to the field. Such studies would enable the approximately 60,000 community pharmacies nationwide to implement more detailed COVID-19 measures and further strengthen the role of community pharmacies in public health. Whereas it is admittedly difficult for community pharmacies to disseminate epidemiological information on their own, it is possible to do so by collaborating with universities and other institutions.

### Limitations

Several limitations exist in this study. Participants were restricted to pharmacists who were managers working in HSPs. Therefore, their responses may not reflect the views or experiences of general pharmacists who work in community pharmacies. There is a possibility that the work description including the chance of the consultation to residents in communities among pharmacy manager and pharmacy manager who own pharmacies somewhat different. These differences may have influenced to the results of the present survey. The conditions during the COVID-19 pandemic have been changing rapidly; thus, community pharmacists’ views regarding the importance of COVID-19 may also have changed since they completed the present survey. Despite these limitations, this was the first KAP survey regarding COVID-19 conducted in Japan that focused on the role of community pharmacists and their interactions with local residents. The findings of this study can contribute to enhancing pharmacists’ roles in their local communities. Further research is warranted, which includes pharmacists working in general community pharmacies.

## Conclusion

Community pharmacists need to deepen their knowledge and understanding of COVID-19 using academic information, such as from scientific papers. This may contribute the prevention of infection with and disease severity of COVID-19 among local residents under the challenging conditions of receiving medical care in a hospital during the COVID-19 pandemic. Such efforts will lead to expansion of the role of community pharmacists as a health partner for local residents.

## Supporting information

S1 Fig COVID-19 consultations provided to local residents at pharmacies, by infection risk area. (TIF)

S2 Fig Main COVID-19 information resources among pharmacists working in health support pharmacies, by infection risk area. (TIF)

S1 Appendix Questionnaire used in this study. (PDF)

S2 Appendix Dataset for the study. (XLSX)
