# Supplementary figures and images for: Knowledge, attitudes, and practices regarding COVID-19 among pharmacists partnering with community residents: A national survey in Japan
Source: PLoS One. 2021 Oct 26;16(10):e0258805. doi: 10.1371/journal.pone.0258805 (PMC8547690; doi:10.1371/journal.pone.0258805)

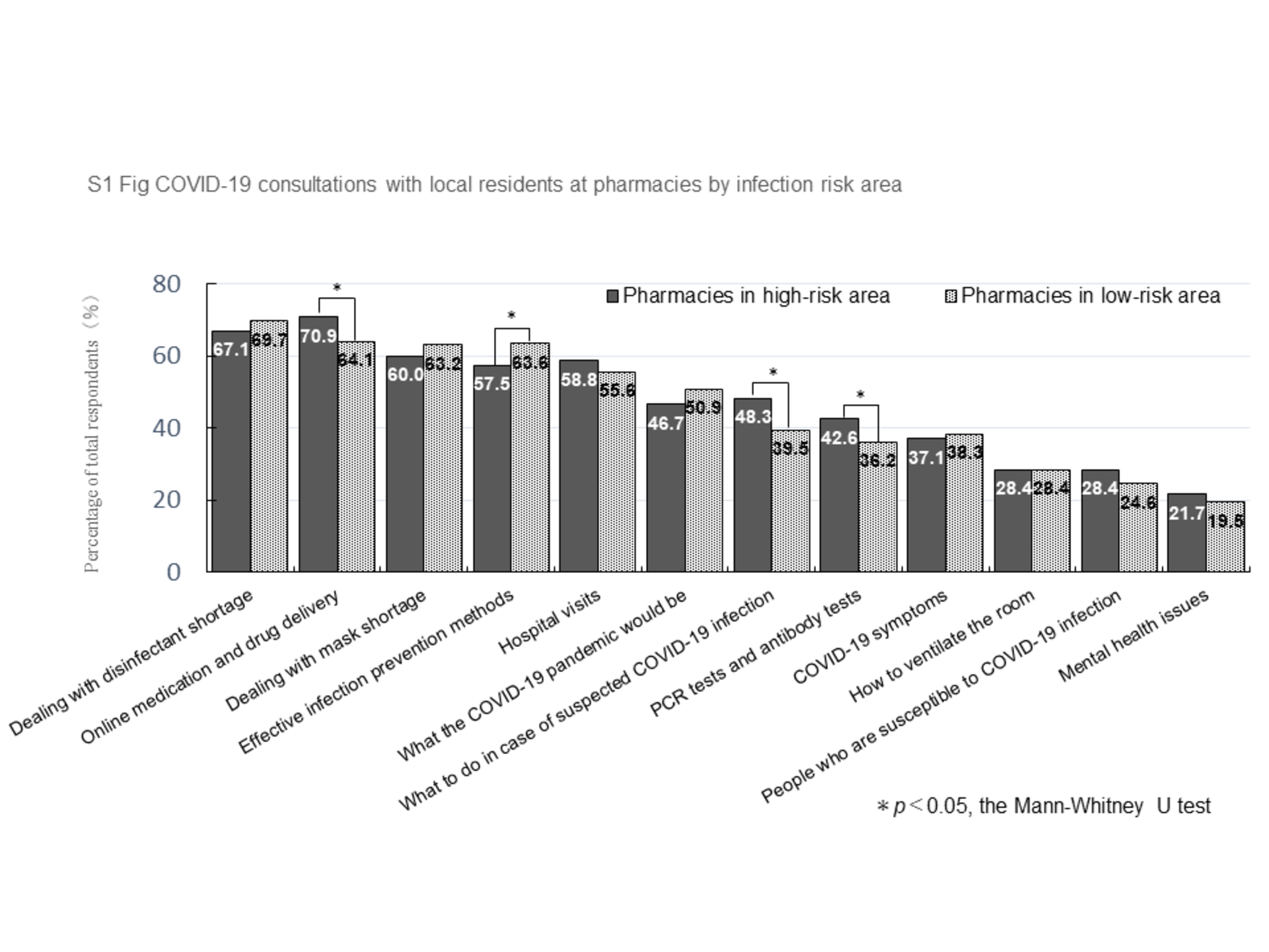

Supplement: S1 Fig — (TIF) [file pone.0258805.s001.tif]

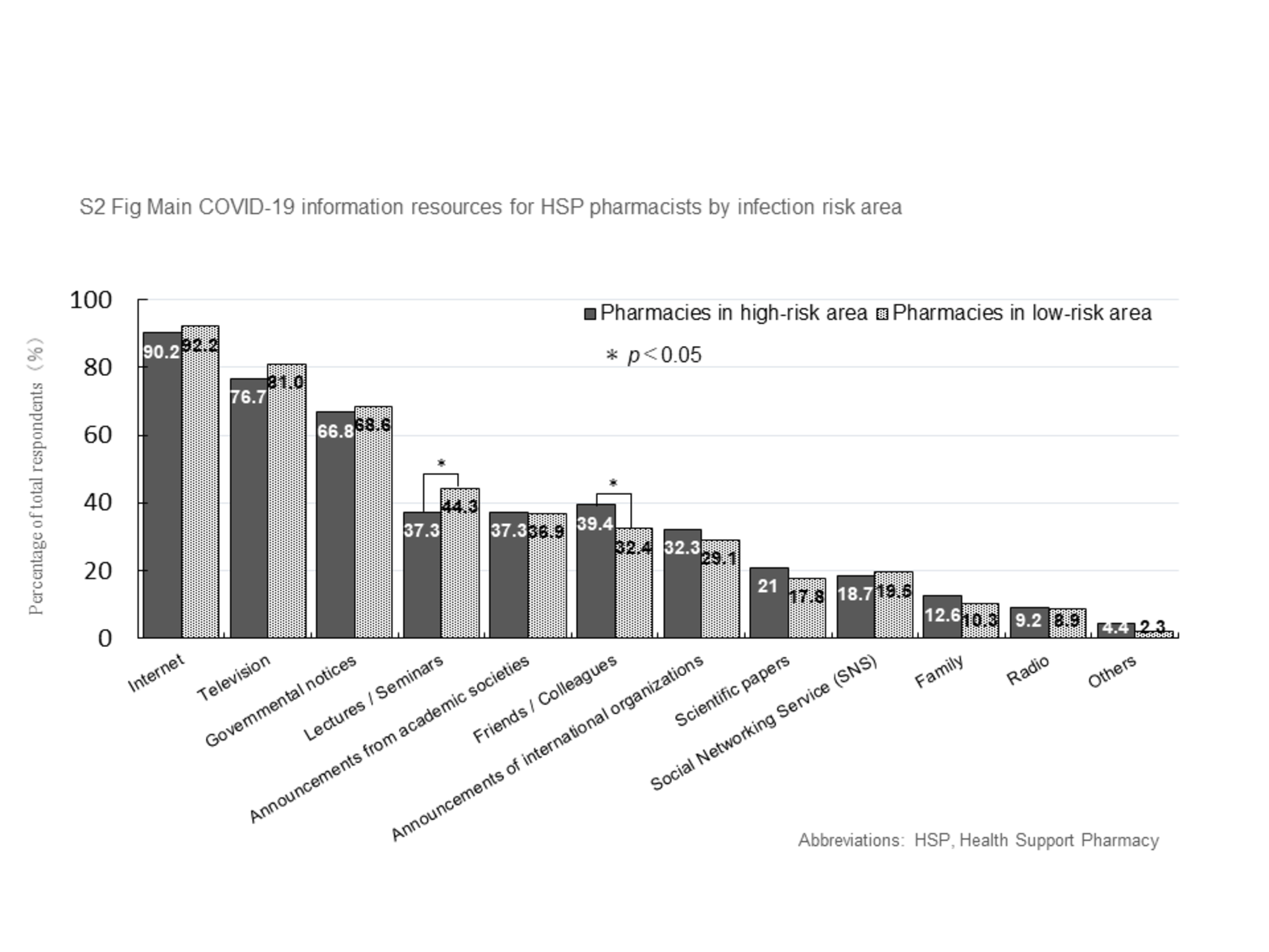

Supplement: S2 Fig — (TIF) [file pone.0258805.s002.tif]
