# Supplementary material for: Knowledge, attitudes, and practices regarding COVID-19 among pharmacists partnering with community residents: A national survey in Japan
Source: PLoS One. 2021 Oct 26;16(10):e0258805. doi: 10.1371/journal.pone.0258805 (PMC8547690; doi:10.1371/journal.pone.0258805)
Supplement: S1 Appendix — (PDF) [file pone.0258805.s003.pdf]

S1 Appendix. Questionnaire used in this study

Q1 Sex

1. Male
2. Female

Q2 Age

Q3 Do you have enough knowledge about COVID-19?

1. Yes
2. No
3. Do not know

Q4 What topics regarding COVID-19 have you discussed in consultations? (Multiple answers allowed)

1. Prediction of situation of COVID-19
2. COVID-19 symptoms
3. People who are susceptible to COVID-19 infection
4. Effective infection prevention methods
5. PCR tests and antibody tests
6. Dealing with mask shortage
7. Dealing with disinfectant shortage
8. What to do in case of suspected COVID-19 infection
9. How to ventilate the room
10. How to exercise
11. Correspondence of breeding animals
12. Good diet to prevent infection
13. Whether to eat raw fish is safe
14. Whether eating alone is better
15. Things to watch out for when shopping
16. Counseling on lack of sleep
17. Mental health issues
18. Hospital visits
19. Online medication and drug delivery
20. No consultations
21. Other

Q5 What COVID-19-related information is needed but is not available? (Multiple answers allowed)

1. Therapeutic drugs and vaccines
2. Infection prevention
3. Post-infection pathology
4. Test information such as PCR and antibody tests
5. Mental health care for patients and medical staff
6. Specific measures to be taken when pharmacy staff are infected
7. Where to find trustworthy information resources
8. Other

Q6 What information do you need to coexist with COVID-19 in the future? (Multiple answers allowed)

1. Accurate epidemiological data
2. Predicting the spread or resolution of infections
3. Infected areas
4. Contact information in case of suspected infection
5. Where to get a PCR test
6. Countries where overseas travel is possible
7. Websites that can collect both medical and pharmaceutical information
8. Materials and handouts for use in pharmacies
9. Mental health measures
10. Other

Q7 What to watch for in clients suspected of having COVID-19 infection in the home (Multiple answers allowed)

1. Separate rooms for infected people
2. Limit the number of people caring for the infected person
3. Make everyone wear a mask
4. Gargle and wash hands frequently
5. Ventilate as much as possible
6. Disinfect common areas
7. Wash soiled linens and clothes
8. Dispose of trash in a sealed container
9. Other

Q8 What I know about COVID-19 (Multiple answers allowed)

1. I do not know anything about COVID-19
2. Required infection protection measures
3. Symptoms of infected people
4. Routes of infection
5. Actions to be taken at the onset of infection
6. Knowing about there are asymptomatic cases of COVID-19
7. Risks of symptom aggravation
8. Required medical care system for COVID-19 patients
9. Mortality risk
10. Consultation counter for COVID-19

Q9 Main resources for COVID-19 information (Multiple answers allowed)

1. Television
2. Radio
3. Internet
4. Social Networking Service (SNS)
5. Family
6. Friends / Colleagues
7. Lectures / Seminars
8. Announcements from academic societies
9. Scientific papers
10. Governmental notices
11. Announcements of international organizations
12. Monks / Priests / Pastors
13. Community Leaders
14. Other

Q10 Trusted resources for COVID-19 information (Multiple answers allowed)

1. Television
2. Radio
3. Internet
4. Social Networking Service (SNS)
5. Family
6. Friends / Colleagues
7. Lectures / Seminars

8. Announcements from academic societies
9. Scientific papers
10. Governmental notices
11. Announcements of international organizations
12. Monks / Priests / Pastors
13. Community Leaders
14. Other

Q11 My job puts me at high risk for COVID-19 infection

1. Agree
2. Disagree
3. Neither agree / disagree

Q12 Who is at high risk for COVID-19 infection? (Multiple answers allowed)

1. Preschooler
2. Primary or junior high school students
3. Young people
4. Adults
5. Elderly people
6. Pregnant women
7. Health care workers

Q13 I think I might be infected with COVID-19

1. Yes
2. No

Q14 How does COVID-19 infection spread? (Multiple answers allowed)

1. Blood-borne infection
2. Coughing and sneezing of infected people
3. Airborne infection
4. Direct contact with an infected person
5. Touching contaminated objects / surfaces
6. Sexual intercourse
7. Contact with contaminated animals
8. Mosquito bites
9. Meals served at a buffet

10. Do not know

Q15 Main symptoms that may be caused by COVID-19? (Multiple answers allowed)

1. Fever
2. Cough
3. Dyspnea / Difficulty breathing
4. Dysgeusia and olfactory dysfunction
5. Muscle pain
6. Headache
7. Diarrhea
8. Malaise
9. No special symptoms
10. Do not know
11. Other

Q16 There are no antiviral drugs that are effective against COVID-19

1. Yes
2. No
3. Do not know

Q17 What is the right way to prevent COVID-19? (Multiple answers allowed)

1. Try to avoid mosquito bites
2. Wash hands regularly using soap and water
3. Eat eggs that are well cooked
4. Wear a mask when in public
5. Avoid close contact with anyone who has a fever
6. Ventilate the room regularly
7. Stay in your room as long as possible
8. Avoid contact with dogs and cats
9. Maintain a nutritionally balanced diet
10. Try not to eat raw fish
11. Get enough sleep
12. Always eat alone
13. Do not know
14. Other

Q18 How much of a threat is COVID-19?

1. A serious threat
2. A neutral threat
3. Not a threat
4. Other

Q19 Factors that make COVID-19 feel threatening (Multiple answers allowed)

1. It does not feel threatening
2. I might become infected
3. I might infect others
4. I might die
5. If I get infected, I might be quarantined
6. My work and daily life will be restricted
7. There is no effective medicine or vaccine
8. Do not know
9. Other

Q20 I will feel embarrassed if I become infected with COVID-19

1. Yes
2. No

Q21 What do you worry about if you became infected with COVID-19? (Multiple answers allowed)

1. Prejudice and discrimination in my neighborhood and workplace
2. Causing trouble in the workplace
3. Family will be subjected to prejudice or discrimination in school or the workplace
4. I may not heal (I will die)
5. Passing the infection on to someone else
6. Causing outbreaks of infectious diseases
7. Decreased income owing to leave of absence
8. Payment for medical treatment
9. Having no one to entrust my work to while recuperating
10. Having no one to delegate household chores to while recuperating
11. Having no one to entrust the long-term care of my family to while recuperating
12. Other

Q22 Things that have influenced your own precautionary behavior (Multiple answers allowed)

1. Government state of emergency
2. WHO Pandemic Declaration
3. Announcement of postponement of the Tokyo Olympics
4. Celebrity deaths from COVID-19 infection
5. COVID-19 infection in people close to me
6. Deaths of people close to me from COVID-19 infection
7. COVID-19 infection in celebrities
8. Other

Q23 I think that the COVID-19 situation will be resolved in the near future

1. Agree
2. Disagree
3. Neither agree / disagree

Q24 What if you or your family have symptoms that suggest COVID-19 infection? (Multiple answers allowed)

1. Seek medical attention immediately
2. Call the health center for advice
3. Contact the workplace
4. Talk to family
5. Talk to a friend or colleague
6. Consult the family doctor
7. Go to a pharmacy or drug store to buy medicine
8. Go to the pharmacy and consult a pharmacist
9. Do not tell anyone
10. Do not go out of the house
11. Other

Q25 Infection measures I have in place for COVID-19 (Multiple answers allowed)

1. Wear a mask where there are other people
2. Wash hands regularly using alcohol-based hand sanitizer or soap and water
3. Gargle
4. Ventilate the room regularly
5. Room cleaning and disinfection
6. Refrain from going out on holiday
7. Avoid contact with people as much as possible

8. Maintain a nutritionally balanced diet
9. Get enough sleep
10. I do not do anything
11. Other

Q26 The infection control measures in the pharmacy where I work are sufficient.

1. Agree
2. Disagree
3. Neither agree / disagree

Q27 What are the concerns about the spread of COVID-19 infection when working at the pharmacy? (Multiple answers allowed)

1. Risk of being infected
2. Securing masks and disinfectants
3. Securing medicines
4. Maintaining operational staff
5. Workload owing to pharmaceutical deliveries
6. Decrease in visiting patients
7. Infection prevention measures in the pharmacy
8. Lack of information related to COVID-19
9. Workload from increase in long-term prescriptions
10. Other

Q28 Infection control measures implemented in the pharmacy (Multiple answers allowed)

1. Disinfect indoor items and equipment
2. Ventilate the room regularly
3. Alert and educate patients about COVID-19
4. Limit the number of patients allowed into the pharmacy
5. Thorough hand washing and masks for pharmacy staff
6. Remove common items, such as magazines and books
7. Encourage cashless payments
8. Offer recommendations for how to request medications online
9. Install shields to protect against infection
10. I do not do anything
11. Other

Q29 I want to keep my current job despite the high risk of COVID-19 infection

1. Yes
2. No

Q30 I am proud of my job

1. Yes
2. No

Q31 I get the flu vaccine every year

1. Yes
2. No
3. Sometimes

Q32 Were you in the habit of wearing a mask even before the COVID-19 pandemic?

1. Yes
2. Only in the cold and flu season
3. No

Q33 Were hand washing and hand hygiene practices in place before the COVID-19 pandemic?

1. Yes
2. Only in the cold and flu season
3. No

Q34 What I was doing to prevent the flu (Multiple answers allowed)

1. Wore a mask around other people
2. Washed my hands and gargled after going out
3. Followed good coughing etiquette
4. Avoided places where people gather as much as possible
5. Refrained from business trips and travel
6. Received influenza vaccination
7. Other

Q35 COVID-19 is a preventable disease

1. Agree
2. Disagree
3. Neither agree / disagree

Q36 Influenza is a preventable disease

1. Agree
2. Disagree
3. Neither agree / disagree

Q37 My academic background

1. 4-year university
2. 6-year university
3. Master’s degree
4. Doctoral degree

Q38 Number of pharmacies owned by the company I work for

1. 1 to 5 pharmacies
2. 6 to 50 pharmacies
3. > 50 pharmacies

Q39 Number of pharmacist coworkers

1. None
2. 2 pharmacists
3. 3 pharmacists
4. 4 pharmacists
5. 5 pharmacists
6. ≥ 6 pharmacists

Q40 Years of experience as a pharmacist

Q41 Number of family members

1. Living alone
2. 2 people
3. 3 people
4. 4 people
5. 5 people
6. ≥ 6 people

Q42 People living in the same household

1. Living alone
2. Living with a spouse
3. Living with spouse and children or grandchildren
4. No spouse, living with children and grandchildren
5. Living with parents and grandparents
6. Living with three generations
7. Other

Q43 Are you caring for a family member?

1. Yes
2. No

Q44 Do you have an underlying disease? (Multiple answers allowed)

1. None
2. Cerebral infarction
3. Hypertension
4. Angina / Arrhythmia
5. Diabetes
6. COPD / Asthma
7. Chronic renal disease
8. Chronic liver disease
9. Other

Q45 I have participated in academic society events, workshops, and study sessions on COVID-19

1. Yes
2. No

Q46 I am involved in the treatment and care of patients with COVID-19

1. Yes
2. No

S1 Appendix (Japanese Questionnaire)
質問票

Q1 性別
1. 男性
2. 女性

Q2 年齢

Q3 COVID-19 について、あなたは充分な情報を持っているか？
1. 充分な情報を持っている
2. 充分ではない
3. わからない

Q4 COVID-19 に対する相談は、どのようなものがあったか？（複数選択）
1. COVID-19 の流行は今後どうなるのか
2. COVID-19 感染したらどうなるのか
3. COVID-19 に感染しやすい人は？
4. COVID-19 の効果的な予防方法
5. PCR 検査、抗体検査について
6. マスク不足の対応方法
7. 消毒薬不足の対応方法
8. COVID-19 感染が疑わしいときの対応
9. 部屋の換気について
10. 運動の方法について
11. 飼っている動物との対処法
12. どのような食事を摂るのが良いのか
13. 刺身など生魚は食べて良いのか
14. 食事は 1 人で摂るのが良いのか
15. 買い物で気を付けることは何か
16. 睡眠不足の相談
17. メンタルヘルスに関すること
18. 病院受診に関する相談
19. 0410 通知対応（オンライン服薬指導・医薬品の配送等）
20. 特に相談はない
21. その他

Q5 COVID-19 について、必要だけど得られないと感じる情報は？（複数選択）

1. 治療薬やワクチンに関する情報
2. 感染予防の為に必要な情報
3. COVID-19 感染後の病態の情報
4. PCR や抗体検査等の検査に関する情報
5. 患者・医療スタッフのメンタルケア
6. 薬局スタッフ感染時の具体的対応法
7. 信頼できる情報源の入手先
8. その他

Q6 これから COVID-19 と共存していく上で、今後必要となってくる情報は？（複数選択）

1. 正確な疫学データ
2. 感染拡大または収束の予測
3. 感染の流行している地域
4. 感染が疑われた場合の問い合わせ窓口
5. PCR 検査を受けられる場所
6. 海外旅行可能国
7. 医学薬学的情報を一辺に収集できるウェブサイト
8. 薬局で使用できる資材・配布資料
9. メンタルヘルスに関する対応法
10. その他

Q7 COVID-19 の感染が家庭内で疑われる来局者へ、どのような注意を行うか？（複数選択）

1. 感染者と他の同居者の部屋を可能な限り分ける
2. 感染者の世話をする人は、出来るだけ限られた方にする
3. できるだけ全員がマスクを使用する
4. こまめにうがい、手洗いする
5. 日中はできるだけ換気をする
6. 取っ手、ノブなどの共用する部分を消毒する
7. 汚れたリネン、衣服を洗濯する
8. ゴミは密閉して捨てる
9. その他

Q8 COVID-19 についてどのようなことを知っているか？（複数選択）

1. 何も知らない

2. 必要な感染防護策について
3. 感染者の症状
4. 感染ルート
5. 発症した場合のとるべき行動
6. 無症状者がいることについて
7. 重症化リスクについて
8.　必要な診療体制
9. 死亡リスクについて
10. 帰国者・接触者相談センターについて

Q9 COVID-19 感染について、主な情報源は何か？（複数選択）
1. テレビ
2. ラジオ
3. インターネット
4. SNS（ソーシャルネットワークサービス）
5. 家族
6. 友人/職場の同僚
7. 講演会／研修会
8. 所属学会等からのアナウンス等
9. 学術論文
10. 行政からの通知やニュースレター
11. WHO や CDC 等の国際機関のアナウンス等
12. 僧侶、神主、牧師等
13. 地域のリーダー（町長、自治会長等）
14. その他

Q10 COVID-19 感染について、何から得た情報を信用するか？（複数選択）
1. テレビ
2. ラジオ
3. インターネット
4. SNS（ソーシャルネットワークサービス）
5. 家族
6. 友人/職場の同僚
7. 講演会／研修会
8. 所属学会等からのアナウンス等
9. 学術論文

10. 行政からの通知やニュースレター
11. WHO や CDC 等の国際機関のアナウンス等
12. 僧侶、神主、牧師等
13. 地域のリーダー（町長、自治会長等）
14. その他

Q11 自分の仕事は COVID-19 に感染リスクの高い職種だと思うか？
1. はい
2. いいえ
3. わからない

Q12 COVID-19 の感染リスクが高いのは誰か？（複数選択）
1. ５歳以下の小児
2. １５歳までの青年
3. 若者
4. 成人
5. 高齢者
6. 妊婦
7. 医療や介護スタッフ

Q13 あなたは COVID-19 に感染する可能性があると思うか？
1. はい
2. いいえ

Q14 COVID-19 は、どのように感染するか？（感染の伝播方法として考えられる全てを選択）
1. 血液から感染
2. 咳やくしゃみなどの飛沫から感染
3. 空気感染
4. 感染者に直接触れる
5. 大勢の人が触る手すり、電車やバスのつり革、ドアノブなど
6. 性交渉
7. 感染した動物との接触
8. 蚊に刺される
9. バイキングで提供された食事
10. わからない

Q15 COVID-19 に感染した場合の症状は？(複数選択)
1. 発熱
2. 咳
3. 息苦しさ、呼吸困難
4. 味覚・嗅覚障害
5. 筋肉痛
6. 頭痛
7. 下痢
8. 倦怠感
9. 特別な症状はない
10. わからない
11. その他

Q16 現在のところ、COVID-19 の原因ウイルス(SARS-CoV-2)に効果のある抗ウイルス薬はない。
1. 正しい
2. 間違い
3. わからない

Q17 COVID-19 の予防方法として、正しいと思われるものは？（複数選択）
1. 蚊にさされないようにする
2. 石鹸や水道から流れる水を使った丹念な手洗いを心掛ける
3. 卵は良く火を通して食べるようにする
4. 人がいるところではマスクをつける
5. 熱がある人との直接接触はなるべく避ける
6. 部屋の換気を心掛ける
7. なるべく部屋の中でじっとしている
8. 犬や猫との接触を避ける
9. 栄養バランスの良い食事を心がける
10. 刺身など生魚は食べないようにする
11. 睡眠を十分にとる
12. 食事は必ず一人でとる
13. 予防方法など何も知らない
14. その他

Q18 COVID-19 感染はあなたにとってどれだけの脅威か？

1. 大変な脅威である
2. 脅威である
3. それほど脅威ではない
4. その他

Q19 COVID-19 があなたにとって脅威となる要因は何か？（複数選択）
1. 脅威ではない
2. 自分が感染するかもしれない
3. 人に感染をうつすかもしれない
4. 死ぬかもしれない
5. 感染したら隔離されるかもしれない
6. 仕事や生活が制限される
7. 現在のところ有効な抗ウイルス薬やワクチンがない
8. わからない
9. その他

Q20 もしもあなたが COVID-19 に感染したら恥ずかしいと思うか？
1. はい
2. いいえ

Q21 もしもあなたが COVID-19 に感染した場合、次のなかで心配される事項は何か？（複数選択）
1. 近所や職場での偏見や差別
2. 職場に迷惑をかける
3. 家族が学校や職場で偏見や差別をうける
4. 治らない（死んでしまう）のではないか
5. 誰かに感染をうつしていないか
6. 集団発生の原因となるのではないか
7. 治療の為の休職に伴う収入減
8. 治療費
9. 療養中、仕事を任せる人がいない
10. 療養中、家事を任せる人がいない
11. 療養中、介護中の親等の面倒を任せる人がいない
12. その他

Q22 あなた自身の"自粛"に影響を与えた事柄は何ですか？（複数選択）

1. 政府の緊急事態宣言
2. WHO のパンデミック宣言
3. オリンピックの延期発表
4. 芸能人やスポーツ選手など有名人の死亡
5. あなたに近い人の感染
6. あなたに近い人の新型コロナによる死亡
7. 芸能人やスポーツ選手など有名人の感染報道
8. その他

Q23 最終的に COVID-19 は収束すると思うか？
1. 収束する
2. 収束しない
3. わからない

Q24 もしあなた又は家族に COVID-19 と思われる症状が出現したら、どうするか？ （複数選択）
1. すぐに医療機関を受診する
2. 保健所に電話をかけて相談する
3. 職場に連絡する
4. 家族に相談する
5. 友人や同僚に相談する
6. かかりつけ医や施設内の医師に相談する
7. 薬局やドラッグストアへ行き薬を買う
8. 薬局に行って薬剤師に相談する
9. 誰にも話さない
10. 家から外に出ないようにする
11. その他

Q25 あなたが実施している自分の COVID-19 感染対策は？（複数選択）
1. マスクの着用
2. 手指衛生
3. うがいの励行
4. 部屋の換気
5. 部屋の掃除・消毒
6. 休日等の外出を控える
7. 可能な限り人との接触を避ける

8. 栄養バランスの良い食事を心がける
9. 睡眠を十分にとる
10. 特に何もしていない
11. その他

Q26 あなたの働く施設の感染対策は十分であると思うか？
1. はい
2. いいえ
3. わからない

Q27 COVID-19 の感染拡大による、あなたの働く施設での不安は何か？（複数選択）
1. COVID-19 の感染リスク
2. マスクや消毒薬の確保
3. 医薬品の確保
4. 運営スタッフの維持
5. 医薬品配送による業務負担
6. 来局患者の減少
7. 薬局内の感染防止対策
8. COVID-19 関連する情報不足
9. 長期処方の増加による業務負担
10. その他

Q28 施設内の感染防御対策として行っていることは？（複数選択可）
1. 室内や器具等の消毒
2. 室内の換気
3. 患者さんらへの注意喚起や教育
4. 薬局内の入場制限
5. 薬局スタッフの感染対策（マスク、手洗い）の徹底
6. 雑誌や絵本、玩具等の共用物の撤去
7. キャッシュレス決済の支払いを推奨
8. オンライン服薬指導の推奨
9. 感染制御のためのシールド設置
10. 特に何もしていない
11. その他

Q29 COVID-19 感染リスクが高くても今の仕事を続けたいか？

1. はい
2. いいえ

Q30 あなたはご自身のお仕事に誇りを持っているか？

1. はい
2. いいえ

Q31 毎年、インフルエンザワクチンを接種しているか？

1. 接種する
2. 接種しない
3. 毎年でなく時々

Q32 COVID-19 パンデミック発生前からマスクをする習慣はあったか？

1. 外出時にはマスクをしていた
2. インフルエンザや感冒のシーズンにはマスクをしていた
3. マスクをする習慣はなかった

Q33 COVID-19 パンデミック発生前から外出後手洗いをする習慣（手指衛生を気にする習慣）はあったか？

1. 手洗い習慣はあった
2. インフルエンザや感冒のシーズンには手指衛生に気を付けていた
3. 手洗いの習慣はなかった

Q34 インフルエンザ感染予防として自分がこれまで実行していたことは何か？（複数選択）

1. マスクの着用
2. 外出後の手洗い・うがい
3. 咳エチケット
4. 人が集まる所に極力行かない
5. 出張や旅行などを控える
6. ワクチンの接種
7. その他

Q35 COVID-19 は予防可能な病気と思うか？

1. はい
2. いいえ
3. わからない

Q36 インフルエンザは予防可能な病気と思うか？
1. はい
2. いいえ
3. わからない

Q37 最終学歴は
1. 4 年制大学
2. 6 年制大学
3. 大学院修士課程
4. 大学院博士課程

Q38 薬局（会社）規模は
1. 1〜5 店舗まで
2. 6〜50 店舗まで
3. 50 店舗より多い

Q39 薬局の薬剤師は自分を含めて何人か？
1. 一人
2. 二人
3. 三人
4. 四人
5. 五人
6. 六人以上

Q40 薬剤師としての勤務年数は？

Q41 同居の家族はご自分を含めて何人か？
1. 一人
2. 二人
3. 三人
4. 四人
5. 五人
6. 六人以上

Q42 同居の家族構成は？
1. 一人暮らし
2. 配偶者と二人暮らし

3. 配偶者と子供や孫
4. 配偶者はいないが子供や孫と同居
5. 親、祖父母と同居
6. 親・祖父母及び子供や孫と同居
7. その他

Q43 現在、家族等の介護をしているか？（仕事による介護を除く）
1. していない
2. している

Q44 現在治療中の病気や怪我はあるか？（複数選択）
1. 特にない
2. 脳梗塞
3. 高血圧
4. 狭心症・不整脈
5. 糖尿病
6. COPD 又は喘息
7. 慢性腎疾患
8. 慢性肝疾患
9. その他

Q45 これまで COVID-19 に関する学会、研修会、講演会等に参加したことはあるか？
1. はい
2. いいえ

Q46 あなたは COVID-19 の患者の治療・介護に関わった（関わっている）か？
1. はい
2. いいえ
